# A Scalable Distribution Network Risk Evaluation Framework via Symbolic Dynamics

**DOI:** 10.1371/journal.pone.0112940

**Published:** 2015-03-19

**Authors:** Kai Yuan, Jian Liu, Kaipei Liu, Tianyuan Tan

**Affiliations:** 1 Reliability Evaluation of Power Systems Group, School of Electrical Engineering, Wuhan University, Hubei, China; 2 Distribution Network Risk Evaluation and Reliability and Life-Cycle Cost Research Unit, School of Electrical Engineering, Wuhan University, Hubei, China; 3 Distribution Network Power Quality Problem Group, School of Electrical Engineering, Wuhan University, Hubei, China; 4 Distribution Network Risk Evaluation and Reliability Research Team, School of Electrical Engineering, Wuhan University, Hubei, China; University of Zurich, SWITZERLAND

## Abstract

**Background:**

Evaluations of electric power distribution network risks must address the problems of incomplete information and changing dynamics. A risk evaluation framework should be adaptable to a specific situation and an evolving understanding of risk.

**Methods:**

This study investigates the use of symbolic dynamics to abstract raw data. After introducing symbolic dynamics operators, Kolmogorov-Sinai entropy and Kullback-Leibler relative entropy are used to quantitatively evaluate relationships between risk sub-factors and main factors. For layered risk indicators, where the factors are categorized into four main factors – device, structure, load and special operation – a merging algorithm using operators to calculate the risk factors is discussed. Finally, an example from the Sanya Power Company is given to demonstrate the feasibility of the proposed method.

**Conclusion:**

Distribution networks are exposed and can be affected by many things. The topology and the operating mode of a distribution network are dynamic, so the faults and their consequences are probabilistic.

## Introduction

Electric power distribution networks are receiving greater attention both from administrators and end users in China as new construction of rural networks and smart grids proceeds.

A distribution network consists of a large number and variety of devices, which are prone to external disruptions. These networks are complex systems, and it is impossible to collect all of the required information for all possible states.

Risk analysis can evaluate both the likelihood of faults occurring and the consequences, which is the traditional concept of reliability used in China. For distribution networks, the probability of faults is related to reliability, which has been the traditional focus of management in power companies. The consequences of faults, however, are usually measured in terms of power loss, which is insufficient.

Risk management has long been a topic of interest both inside and outside the power industry. Several researchers have focused on distribution network investment. Under brink attempted to relate component failures and repair times to power losses [1].Sand and others attempted to improve maintenance and reinvestment decisions through Bayesian networks by correlating certain variable indicators such as adverse weather with risk [2–4]. Janjicat tempted to decouple risk factors and state transitions based on decision tree diagrams and then adjust maintenance schedules [5].Risk-based management has been used in many aspects of power system planning [6–9].

Because a distribution network is a large-scale system, the availability of power is influenced by component reliability, the network structure, maintenance, the operating condition, the environment and other factors. Risk analysis based on component failures or system failures cannot include all of the factors affecting distribution network risk. For example, a transformer failure may be the result of poor quality manufacturing, a lightning strike, poor maintenance, prolonged overloaded operation or other reasons. Risk analysis based on the failure time would miss these details, which would be very important for risk reduction decisions.

Consequences in risk analysis cannot be simply measured or converted to failure times because this would not reflect all of the loss characteristics. For example, a one-hour power outage would not have the same consequences for a five-star hotel as for a remote village. The expected remedies would also be different, which would result in different investment decisions.

To describe distribution network risk, one-dimensional time series data, which usually fluctuate over time, should be collected from multiple sources. Processing the data using probability theory can reveal the uncertain characteristics of risk[10],e.g., for load forecasting[11]. Xiao developed probabilistic indices and attempted to control for low voltage and overload using a multi-objective approach[12].Feng processed data with a random fuzzy model and evaluated the operation risk[13]. Other researchers have used radial basis function neural networks, hybrid methods, equivalent reliability networks and other methods to simplify the analysis[14–16], but external influences were not included.

For systems with incomplete information, it is logical to consider semi-empirical methods[17]. However, as technology improves and requirements emerge, data may be added, updated or deleted from the system, so a scalable framework for risk analysis is critical.

Symbolic dynamics can be used to analyze one-dimensional time series data, and this method is widely used in anomaly detection and pattern recognition [18–19]. In this study, symbolic dynamics are used to abstract information contained in raw data, and entropy theory is used to analyze risk factor relationships.

## Materials and Methods

### Risk and Symbolic Dynamics

Distribution networks are operated in the open, and they are at risk from a great many factors that are difficult to enumerate. A risk description framework should be adaptable to the current management strategy, the evolution of technology and an understanding of risk for the distribution network.

To describe risk in a distribution network, data of various types such as continuous load data or discrete user-level data should be collected. Current approaches tend to use fuzzy set theory to abstract or categorize data, but the coarse nature of fuzzy sets precludes further processing at a finer granularity.

Quantitatively, risk is defined as:

Risk=Possibility×Loss (1)

Where *Possibility* is the likelihood of the occurrence of a particular fault and *Loss* is the consequence of that fault occurring. Currently, consequences in distribution network risk analysis are mostly measured by power loss, which is inadequate. As an example, a power loss would have different effects, both in economic and social terms, in a five-star hotel and in a rural village. Additionally, a consequence in a distribution network is not static because the topology and the operating mode can change.

For a discrete time series, any set of disjoint regions $\beta ={\left\{C_{i}\right\}}_{1}^{m}$ that covers the state space *S* is called a partition[20]; that is,

$$\beta ={\left\{C_{i}\right\}}_{1}^{m};C_{i}\cap C_{j}=\varphi fori\neq j;\overset{m}{\underset{i=1}{\cup }}C_{i}=S$$ (2)

If a unique symbol *m*∈*Ω*, where *Ω* is a symbol set defined as {*S0*,*S1*,*S2*,*…*,*Sm-1*}, is assigned to a specific partition, then the representation of the time series data would be

$$L_{X}(L,i)=\sum_{p=1}^{L}m^{L-P}S(p+i)$$ (3)

where *i* is the starting index of the symbolic in the symbol set *Ω* and *m* is the length of the symbol sequence. Similar to fuzzy sets, this symbolic representation can abstract the information, but this representation permits more flexibility and uncertainty than fuzzy sets. It is assumed that the dynamical system is stationary on the fast time scale and that any nonstationarity is observable only on the slow time scale. In symbolic dynamics, the *slow time scale* is typically defined as being at least two orders of magnitude larger than the *fast time scale*.

For convenience, we define five levels to describe the risk in the distribution network, *very high*, *high*, *medium*, *low*, and *very low*, which can be represented by a symbol set *Ω* = {*A*,*B*,*C*,*…*,*O*}.

### Risk Description Framework

**Fig 1:**
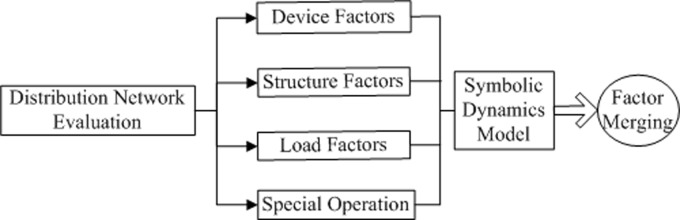
Risk description framework.

We propose a risk description framework that includes device, structure, load and special operation factors, as Fig. 1 illustrates.

**Fig 2:**
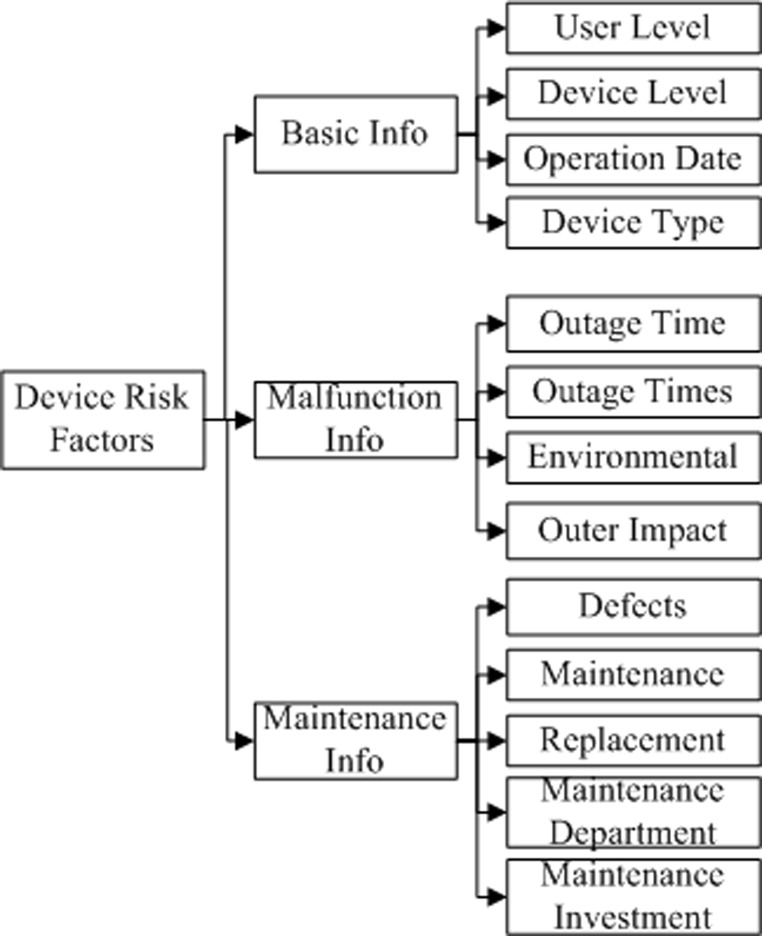
Example of a layered framework for device factors.

All risk indicators should be calculated independently according to the voltage level. For convenience, it is logical to organize the factors in a layered structure. Theoretically, the more data that are collected, the more accurate the evaluation of risk will be. Because the types of data may vary with location and time, the factor merging algorithm should be robust and flexible. As an example, the organization for the device indicators is given in Fig. 2.

The risk factors may also have sub-factors such as environmental effects, but these will not be discussed here. As China covers vast area, it is hard to adopt uniform risk factors framework. Practically, Risk indices selection and categorization is first carried out by national standards. Then, supplementary indices are integrated into the framework according to local data collection ability and management requirement.

### Raw Risk Data Processing

Risk, as defined previously, is a relative value, so a baseline should be chosen for evaluation. For a distribution network risk evaluation, a day with fine weather, a light load, and no defects or malfunctions should be chosen as the baseline. The risk factors can be mapped linearly based on the baseline extreme values. A mapping process is described in the following.

**1. Possibility Data Processing.** For the device factor layers shown in Fig. 2, we calculate the relative level factor for a line or a substation as

$$D_{LevelFactor}=\frac{\sum_{l=1}^{k}Device(l)\times Device_{Level}}{Device_{Count}}$$ (4)

Where *Device*_*Count*_ and *Device*_*Level*_ are self-explanatory and *Device(l)* is the device number at a specific level. After the baseline factor is calculated, a mapping from the raw data to a symbolic sequence can be defined as follows:

$$Ind_{R}=\left\{ \begin{array}{l} \frac{Ind_{Cur}-Ind_{Min}}{\left(Ind_{Max}-Ind_{Min}\right)}\times 100,Ind_{Cur}\leq Ind_{Max}\\ 100,Ind_{Cur}>Ind_{Max} \end{array}\right.$$ (5)

$$P_{Idx}=\left\{ \begin{array}{l} \left[\frac{Ind_{R}}{20}+1\right]\times 3,x-\left[\frac{Ind_{R}}{20}\right]\times 20<12\\ \left[\frac{Ind_{R}}{20}+1\right]\times 3+1,12\leq x-\left[\frac{Ind_{R}}{20}\right]\times 20<18\\ \left[\frac{Ind_{R}}{20}+1\right]\times 3+2,18\leq x-\left[\frac{Ind_{R}}{20}\right]\times 20<20 \end{array}\right.$$ (6)

Where *Ind*_*Max*_ and *Ind*_*Min*_ are the maximum and minimum values of the baseline calculations, respectively, and *P*_*Idx*_ is the first symbol index in the symbol set. We chose three symbols to describe the risk probability and consequence. Other symbols are consecutive symbols after *P*_*Idx*_ indicates. The symbols indicate weights *W*_*s*_ of 60%, 30% and 10%, respectively. The first and second symbol weights are approximation of golden number, and the rest is allocated to the third symbol weight.

**2. Consequence Data Processing.** The risk factors can have either direct or indirect connections to a malfunction. For factors with a direct connection such as a device failure, the mapping is defined as follows:

$$Con_{R}=Min\left\{100,\frac{MTTR_{Idn}}{MTTR_{Avg}}\times Level^{2}\times 100\right\}$$ (7)

$$C_{Idx}=\left\{ \begin{array}{l} \left[\frac{Con_{R}}{20}+1\right]\times 3,x-\left[\frac{Con_{R}}{20}\right]\times 20<12\\ \left[\frac{Con_{R}}{20}+1\right]\times 3+1,12\leq x-\left[\frac{Con_{R}}{20}\right]\times 20<18\\ \left[\frac{Con_{R}}{20}+1\right]\times 3+2,18\leq x-\left[\frac{Con_{R}}{20}\right]\times 20<20 \end{array}\right.$$ (8)

Where *C*_*Idx*_ is similar to *P*_*Idx*_, *MTTR*_*Idn*_ and *MTTR*_*Avg*_ represent the affected factor recovery time and the total line or substation recovery time expressed in terms of *MTTR* (Mean Time To Repair), and *Level* in equation (7) indicates the relative importance of the line or substation.

From equation (7), we observe that the line or substation level has a strong influence on the risk consequence.

For indirect connection factors, we convert the raw data in a relative manner. For example, the maintenance department risk consequence *Con*_*R*_ for a distribution line can be calculated as

$$Con_{R}=Min\left\{100,\frac{MTTR_{IdnMngAvg}}{MTTR_{AvgAll}}\times Level^{2}\times 100\right\}$$ (9)

Where *MTTR*_*IdnMngAvg*_ is the average *MTTR* under a specific management staff and *MTTR*_*AvgAll*_ is the average *MTTR* of all of the lines. The average *MTTR* is calculated from the line *MTTR* and the line length. For example, *MTTR*_*IdnMngAvg*_ is calculated using equation (10)

$$MTTR_{IdnMngAvg}=\frac{MTTR_{Mng}}{Line_{MngLength}}$$ (10)

Where *MTTR*_*Avg*_ includes all of the MTTRs under a specific management staff and *Line*_*MngLength*_ is the corresponding line length.

### Phase-Space Reconstruction

Once the factors in the risk description have been decided, the phase-space dimension and structure are determined. It is possible to recreate the entire trajectory of the system from measurements. Based on equation (6) and the symbol sequence representation, the sequence of state vectors is represented as:

$${\bar{S}}_{0}=\left[\begin{array}{l} Ind_{0}(t)\\ Ind_{1}(t)\\ ...\\ Ind_{m-1}(t) \end{array}\right],...,{\bar{S}}_{i}=\left[\begin{array}{l} Ind_{0}(t+i\Delta t)\\ Ind_{1}(t+i\Delta t)\\ ...\\ Ind_{m-1}(t+i\Delta t) \end{array}\right]$$ (11)

where{*Ind*_*k*_} is the sequence of the state vectors generated from the raw risk data processing and*Δ∈N* is a time interval in the phase-space trajectory of the system determined by the observation rate. To reflect the layered structure of the risk factors, the factors are grouped according to their place in the risk description framework, such as in Fig. 2.

### Symbolic Dynamics Operators

The processing of the raw data and the phase-space reconstruction were discussed in the previous section. The symbolic dynamics operators are presented in this section to establish a foundation for factor merging.

***definition 1***: Sequence Index Operator ***Idx***

The sequence index operator is defined as

$$t=Idx(L_{X}(3))$$ (12)

where*t* is the index of the first symbol in the symbol set *Ω* for a given symbol sequence. The risk probability and consequence are represented by a symbol sequence of 3, *L*_*X*_ *(3)*.

***definition 2***: Shift Operator **→**

The shift operator is defined as

$$L_{X1}(3)=L_{X}(3)\to g$$ (13)

where*g* is an integer indicating the amount of shift in the symbol set *Ω* where positive means a shift to the right and negative means a shift to the left. The shift operation cannot cross the border of the symbol set. If the shift operation reaches the symbol set border, the last symbol should be repeated. For example, if the first index is 14 or 15 and the symbol set is {A, B, …, O}, the result of the shift operator would be *L*_*X*_ *(3)* = {NOO} or *L*_*X*_ *(3)* = {OOO}.

***definition 3***: Addition Operator ⊕ The addition operator is defined as

$$Idx(L_{X1}(3)⊕L_{X2}(3))=[\sum_{l=1}^{3}\begin{array}{l} \left(Idx(L_{X1}(3,l)\times W_{s}(X1,l\right)\\ +Idx(L_{X2}(3,l)\times W_{s}(X2,l)) \end{array}\text{]}$$ (14)

where[] is the Gaussian function and*Ws(X1*,*l)* and *Ws(X2*,*l)* are symbol weights as described in Section II Raw Risk Data Processing. Add operator would get the first symbol index of the result symbol sequence.

***definition 4***: Multiplication Operator ⊗

The multiplication operator is defined as

$$Idx(L_{X1}(3)⊗L_{X2}(3))=[\sum_{l=1}^{3}\begin{array}{l} \left(Idx(L_{X1}(3,l)\times W_{s}(X1,l\right)\\ \times Idx(L_{X2}(3,l)\times W_{s}(X2,l)) \end{array}\text{]}$$ (15)

***definition 5***: Ratio Operator **Θ**

The ratio operator is defined as

$$Idx(L_{X}(3)\Theta x)=x\times [\sum_{l=1}^{3}\left(Idx(L_{X}(3,l)\times W_{s}(l)\right)\text{]}$$ (16)

where *x* is a positive rational number.

As explained in definition 1, the operations in definitions 2–5 cannot exceed the symbol boundaries.

## Results

As mentioned previously, raw risk factor data may vary with location and time. Therefore, it is critical to build a scalable framework. The framework in Fig. 2 is a scalable framework that enables users to add or remove factors as necessary. In this section, the system symbolic description and operators discussed in the previous sections are used to establish an algorithm for distribution network risk evaluation that is scalable.

### Risk Factor Correlation

**Fig 3:**
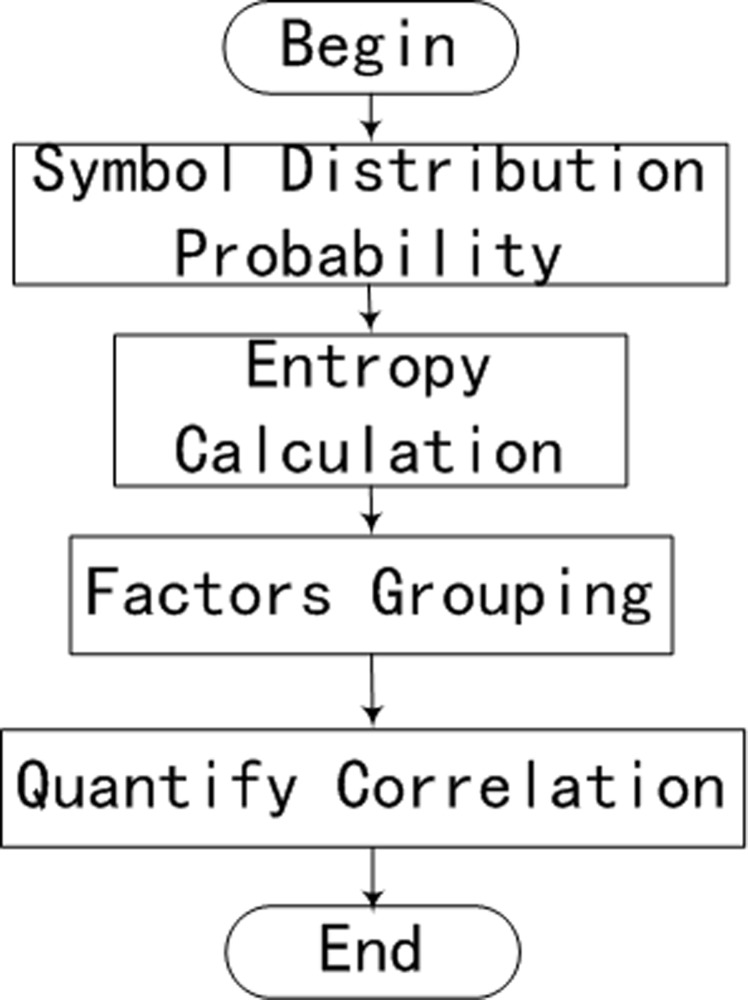
Risk factor correlation flowchart.

In a layered risk evaluation framework, risk sub-factors contribute to higher-layer risk factors. Because sub-factors may have different effects on the main factor, it is important to measure the relationship between the sub-factors and the main factor. A statistical method is used with the assumption that the more information is available, the more accurate the evaluation of risk will be. Therefore, we calculate the correlations of risk factors, as illustrated Fig. 3 and as described in the following.

**1. Symbol Distribution Calculation.** Based on the state vector *{Ind*_*k*_ *}* in equation (11), a symbol *j* in symbol set*Ω*has the distribution probability

$$p_{i}(j)=\frac{\sum_{l=1}^{k}W_{s}(l)}{k\times W_{s}(Max)}$$ (17)

where *k* is the time index of the state vector, if symbol *j* exists, over which the symbol weights are accumulated and *W*_*s*_ *(Max)* is the maximum symbol weight, say 60%.

Then, for a specific risk factor, the symbol distribution probability is calculated. For the main risk factor, the overall symbol distribution probability can be calculated as

$$P_{i}=\frac{\sum_{d=1}^{r}\sum_{l=1}^{k}W_{s}(d,l)}{k\times r\times W_{s}(Max)}$$ (18)

where *r* is the number of main risk factors, *W*_*s*_ *(d*,*l)* is the corresponding *d*th sub-factor symbol weight.

**2. Entropy Calculation.** After the symbol distribution probability has been calculated for the risk sub-factors, likeness for the sub-factors can be analyzed, which is useful for grouping them.

We use Kolmogorov-Sinai entropy, which is defined in equation (19), to measure the randomness of the risk factors, and we use the Kullback-Leibler distance in equation (20) to quantify the likeness of the risk factors.

$$H_{j}(c)=\sum_{c\in \Omega }p_{j}(c)\log_{2}p_{j}(c)$$ (19)

$$D(p_{j}||p_{r})=\sum_{c\in \Omega }\log_{2}(\frac{p_{j}(c)}{p_{r}(c)})$$ (20)

**3. Factor Grouping.** It should be noted that the randomness of the symbol distributions affects the accuracy of the Kullback-Leibler distance. Therefore, we define a refined measurement as

$$I_{i,j}=\frac{D(p_{j}||p_{r})}{H_{i}\times H_{r}}$$ (21)

This measurement was chosen such that, even if the Kullback-Leibler distance is small, a high degree of randomness in the Kolmogorov-Sinai entropy reduces the possibility of two risk factors belonging to the same group, and vice versa. The group threshold is set at 2 to allow the largest possible grouping of similar sub-factors. For a specific main risk factor, its *n* sub-factors are grouped into *f* categories of risk subsets.

The goal is to correlate sub-factors to main factors through the symbol distribution probabilities, but if certain types of data are more abundant than others, the information in the less-abundant data may be obscured. Grouping data into categories not only reduces the dimension of the space, which further simplifies the process, it can reveal information that would otherwise be lost.

**4. Quantification of Correlations.** This step attempts to relate the *f* categories of risk subsets to the main risk factor. We will describe the process using an example.

Without loss of generality, assume that a category *f1∈f* has *m1*sub-factors. We could then construct the time series of *f1*as in equation (11); for example,*S*_*f1*_ *(t)* = [*Ind*_*0*_ *(t)*,*…*,*Ind*_*m(1)-1*_ *(t)*]^T^. For each state vector in the time series, an average operation is defined as

$$Idx(S_{AvgX}(t,3))=[\sum_{l=0}^{m_{\left(1\right)}-1}Ind_{l}\text{(}t\text{)}\Theta \frac{\text{1}}{m_{\left(1\right)}}\text{]}$$ (22)

In this manner, the state vector phase-space is reduced from *m* to *f*. From equations (17)–(21), we can calculate the distance between the *f* categories and the distribution of all the indicators as

$$I_{i}=\frac{D(p_{j}||P)}{H_{i}\times P}$$ (23)

This is the quantitative distance between the sub-factor set and the main risk factor. The quantitative correlation coefficient is defined as

$$\varepsilon_{i}=\frac{I_{i}^{2}\times m_{\left(i\right)}}{{\sum_{t=1}^{n}I}_{t}^{2}\times m_{\left(t\right)}}$$ (24)

This equation indicates that both the Kullback-Leibler distance and the number of factors in a sub-factor set contribute to the correlation coefficient and that the Kullback-Leibler distance has more influence.

### Risk Factor Merging

Merging of risk factors simplifies the calculation of main risk factors from sub-factors. Merging is defined as

$$Idx(L_{MotherX}(t,3))=[(\sum_{l=0}^{n}Ind_{l}\text{(}t\text{)}\Theta \varepsilon_{l}\text{)}\Theta \frac{1}{n}\text{]}$$ (25)

where *n* is the number of sub-factor groups.

### Event Risk Calculation

Prior to this step, the risk is calculated from the failure probability and the consequence independently.

For a specific component, line or substation, we can calculate the overall risk as

$$Idx(L_{Risk}(3))=L_{\mathrm{Pr}obability}(3)⊗L_{Consequence}(3)$$ (26)

However, this definition, which was derived from equation (1), is mainly a statistical result. The variable nature of risk is not included. Therefore, an improvement is desired.

Using the shift operator in definition 2 on the phase-space in equation (11), we can obtain another time series vector for some value of *g*. Referring to the factor grouping and factor merging methods, we can define a fluctuation parameter as

$$P_{f}=[(\sum_{l=0}^{f}\left\|g(l,t)\right\|\times \varepsilon_{l}\text{)}\Theta \frac{1}{f}\text{]}$$ (27)

To merge the four major types of risk factors, we define the merge operation given by equation (28),

Risk=Line(StructIndΘIndSociety)⊗{((DeviceInd⊗LoadInd)ΘIndWeather)⊕TechInd} (28)

where*Device*_*Ind*_, *Struct*_*Ind*_, *Tech*_*Ind*_ and *Load*_*Ind*_ are the device, structure, special operation and load factors, respectively, and *Ind*_*Society*_ and *Ind*_*Weather*_ are social and weather effect parameters, respectively, selected in accordance with the norms of that locality.

The final overall risk is defined as

$$Idx(L_{Risk}(3))=(L_{\mathrm{Pr}obability}(3)⊗L_{Consequence}(3))\Theta P_{f}$$ (29)

This equation shows that greater diversity in the sub-factors results in greater risk of the event.

### Algorithm Discussion

Risk is a relative concept based on probability theory. In distribution networks, if remedial measures and schedule planswere included in the risk evaluation, the failures and the losses would all have probabilistic characteristics. The proposed method is built on symbolic dynamics, and the result is intuitive, which is helpful in management. The following discussion further explains the concepts and the implementation of the method.

**1. Information Abstraction.** A distribution network is a complex dynamic system that involves many types and large volumes of data. Therefore, information abstraction is very important.

Because risk is a relative concept, a linear demarcation of baseline data for basic risk standards as described in equations (5) and (6) is feasible, but this approach is not accurate. Furthermore, because the probabilistic nature of risk leads to vagueness in its evaluation, symbolic dynamics are used to incorporate language vagueness in the risk description.

Compared with fuzzy set abstraction, which expresses a variable using a definite category, symbolic dynamics use a symbol sequence to describe a variable, which enables further information processing.

Because all raw data are mapped into the symbol set, further uniform processing can be achieved. This relative processing technique conforms to the risk concept. The method of merging risk factors using symbolic dynamics operators offers a new way to compute risk factor relationships.

**2. Probability-based Analysis.** Risk is a probability-based concept. For layered risk factors, sub-factors can affect risk factors in higher layers. Under these assumptions, the symbol distributions are calculated to reflect the failure and consequence probabilities. The Kullback-Leibler distance is used to measure the relationships between the sub-factors and the main risk factors and can be used as coefficients to adjust for variable randomness.

**3. Scalability.** The data may vary with location or time, and they may have different numbers of sources. Thus, the risk framework should scale according to the data sources and should not allow data from more sources that is greater in volume to mask information conveyed by data from fewer sources.

Equation (22) provides the mean value to describe a risk factor category, and equation (25) merges risk factor categories. Regardless of the number of sub-factors in a risk category or the number of categories, this method provides a uniform risk value. Therefore, the risk can be calculated for the same description framework regardless of the number of data sources, which permits scalability.

**4. Complexity Analysis.** Table 1 gives the approximate computational complexity estimates for the various steps in the algorithm, assuming the basic dimension of description state vector is *m*, as in equation (11).

**Table 1:** Computational Complexities of Algorithm Steps.

**Step**	**Complexity**
Symbol Distribution	*O(m* ^*2*^ *)*
Entropy Calculation	*O(m)*
Factors Grouping	*O(m* ^*2*^ *)*
Quantify Correlation	*O(m* ^*2*^ *)*
Factors Merging	*O(m)*
Event Risk Calculation	*O(m)*

**5. Algorithm Acceleration.** The most time-consuming processes are the symbol distribution, the factor grouping and the factor merging. Because these operations are based on historical data, they can be performed at system initialization and then updated periodically. In this manner, each step can be reduced to *O(m)*complexity, which is very desirable.

**6. Multiple Granularity Management.** Because the risk factors are layered, grouped and calculated, the risk failure and consequence distributions can be calculated and the correlations between risk factors can be tracked. Therefore, management of risk factors with differing granularities can be implemented. From the risk failure and consequence distributions, proper countermeasures may be taken. From the correlations between risk factors, counter measures can be prioritized.

## Discussion

The Sanya Power Company supplies power to Sanya, a popular tourism site in China. The company controls three 220kV substations, twelve 110kV substations, seven 220kV lines, twenty-four 110kV lines and one hundred and eighty-one 10kV lines. Sanya is a tropical island, so its distribution network is prone to disruptions from weather and other environmental factors. Therefore, risk management is very important for improving reliability.

**Fig 4:**
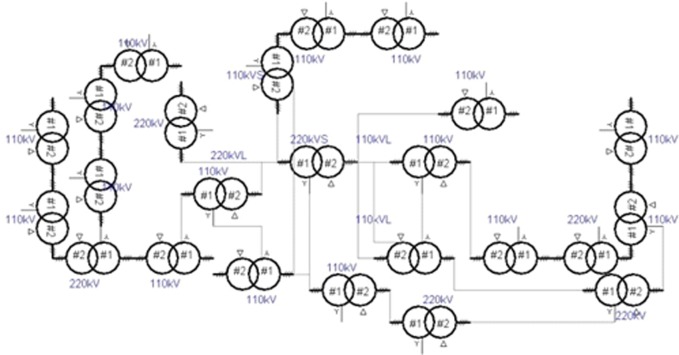
High-voltage distribution network.

Fig. 4 shows the high-voltage distribution network in Sanya. Certain substations or lines that are not under the SPC’s administration are included to simplify the calculations.

The risk was given five levels, as shown in Table 2.

**Table 2:** Risk Level.

**Risk Level**	**Symbol**
Very High(V)	A
High(IV)	D
Medium(III)	G
Low(II)	J
Very Low(I)	M

In our research, following data were collected:

1. Distribution network topology
2. Device accounting
3. Power flow data
4. Deficiencies and malfunctions from 2006 to 2013
5. Device-level and user-level data
6. Distribution network operation reports

Various loads, weather conditions and community activities may affect the overall risk, as equation (28) indicates. However, risk is a relative value. In our evaluation for 2013, the baseline was set at the minimum overall load day in 2009. We will list three line analysis results in this section, namely Yali II, Yali I and Yatian, which are high-risk transmission lines. The results for the substations are omitted for brevity.

In this example, the maximum overall load day and a typical rainy day in 2013were selected. The baseline, the maximum overall load day and the rainy day in 2013were evaluated using the parameters given in Table 3.

**Table 3:** Evaluation Parameters.

**Evaluation Number**	**IndWeather**	**IndSociety**
1	1.0	1.0
2	1.0	1.0
3	2.0	1.4

The sub-factor correlation coefficients obtained from equations (22) to (24)are listed in Table 4.

**Table 4:** Line Device Indicator Correlation Coefficients.

**Correlation**	**Yali II**	**Yali I**	**Yatian**
User Level	4.2	4.1	3.1
Device Level	2	1	1
Operation Date	1.0	1.3	1.5
Device Type	1.0	1.5	1.2
Outage Time	0.8	1.1	1.1
Environmental	1.8	1.9	2.2
Outer Impact	1	1.3	1.2
Defects	1.2	5.9	4.3
Maintenance	1.2	1.7	1.5
Replacement	1.1	1.5	1.3
Maintenance Department	4.2	4.6	4.3
Maintenance Investment	1.2	2.3	2.5

From Table 4, we conclude the following:

1. The user level and the maintenance are highly correlated with the overall risk.
2. Because Yali II is a relatively new line, defect management for that line has less of an effect than it does with the older lines, Yali I and Yatian.

**Fig 5:**
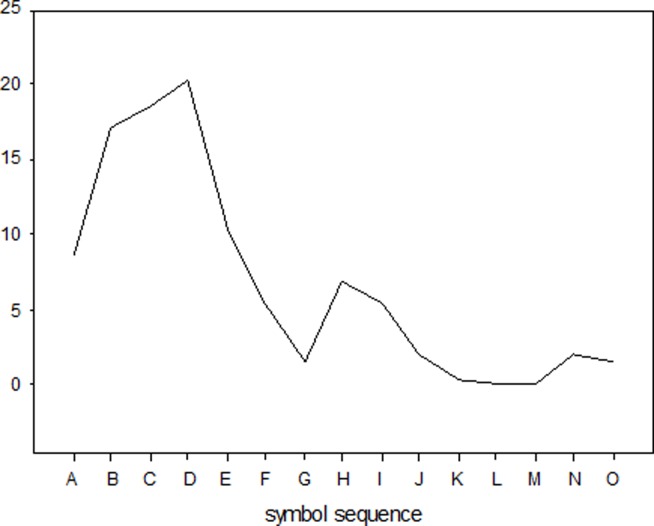
Device failure probability symbol distribution.

**Fig 6:**
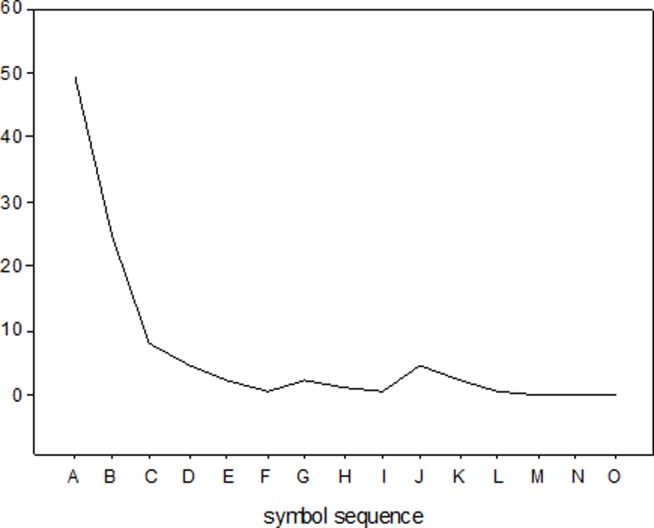
Device consequence probability symbol distribution.

The structure indicator varied insignificantly in our three evaluation examples. Fig. 5 and Fig. 6 give the symbol distributions for a 110kV line structure failure and consequence, respectively. Table 5 lists the corresponding symbol distribution probabilities.

**Table 5:** Symbol Distributions (%).

**Symbol**	**Failure Symbol Distribution**	**Consequence Symbol Distribution**
A	8.57	49.28
B	17.14	24.64
C	18.57	8.21
D	20.35	4.31
E	10.36	2.14
F	5.35	0.71
G	1.43	2.14
H	6.79	1.07
I	5.37	0.36
J	2.14	4.29
K	0.36	2.14
L	0	0.71
M	0	0
N	2.14	0
O	1.43	0

The overall device risk indicators calculated from equations (25) and (26) are listed in Table 6.

**Table 6:** Line Device Risk.

**Line**	**Line Risk Factor Value/Symbol**		
	**Evaluation 1**	**Evaluation 2**	**Evaluation 3**
Yali I	1.52/BCD	1.58/BCD	2.12/CDE
Yali II	1.87/BCD	1.92/BCD	2.42/CDE
Yatian	1.87/BCD	2.01/CDE	2.15/CDE

As can be observed from Table 6, the 110kV line is relatively reliable. A comparison with a high-risk 10kV line is given in Table 7.

**Table 7:** 10kV Line Device Risk.

**Line**	**Line Risk Factor Value/Symbol**		
	**Evaluation 1**	**Evaluation 2**	**Evaluation 3**
DongHaibin II	5.36/HIJ	7.35/KLM	7.35/KLM
Xijin	4.37/GHI	5.68/IJK	5.68/IJK
Dadonghai II	6.16/IJK	8.16/LMN	8.16/LMN

The overall risk values are listed in Table 8. To compare high-voltage risk characteristics, we give the risk analysis results for several10kV lines in Table 9.

**Table 8:** Transmission Line Overall Risk.

**Line Name/Evaluation Number**		**Risk**					
		**Structure**	**Device**	**Operation**	**Load**	**Result**	**Risk Level**
Yali I	1	23.5	1.52	1.5	1.38	84.66	IV
	2	23.5	1.58	1.5	1.46	89.64	IV
	3	23.5	2.12	1.5	1.33	234.86	V
Yali II	1	23.5	1.87	1.0	1.14	73.67	III
	2	23.5	1.92	1.0	1.24	79.34	III
	3	23.5	2.10	1.0	1.09	182.83	V
Yatian	1	14.2	1.87	1.3	1.09	44.97	II
	2	14.2	2.01	1.3	1.08	49.34	II
	3	14.2	2.15	1.3	0.85	98.68	IV

**Table 9:** 10kV Line Overall Risk.

**Line Name/Evaluation Number**	**Risk**						
	**Structure**		**Device**	**Operation**	**Load**	**Result**	**Risk Level**
Tianhai	1	6.91	5.36	3.20	7	281.37	V
	2	8.06	7.35	3.20	12	731.68	V
	3	7.85	7.35	3.20	8	1327.59	V
Gang	1	10	4.37	7.07	3	201.8	V
	2	11.65	5.68	7.08	11	801.37	V
	3	12.38	5.68	7.08	6	1304.06	V
FengH	1	9.84	6.16	3.55	3	216.77	V
	2	13.48	8.16	3.55	6	707.83	V
	3	9.39	8.16	3.55	7	1548.47	V

Although algorithm in this paper has little constraints on data availability. As precision for symbolic dynamics data based abstraction and entropy based correlation evaluation, lack of data would have great impact on the rationality of the result. The more data available, the more precise the result is.

Although the categorization would also influence the final result, data categorization can be carried out under national or provincial system monitor, maintenance guidance, which would leads to uniform categorization in a relatively large area.

## Conclusions

Distribution networks are exposed, and their operation can be disrupted for many reasons. Because the topology and the operating mode of a distribution network are dynamic, failures and their consequence are probabilistic in nature. This study investigated a risk evaluation method based on symbolic dynamics. Because of the relative nature of risk, symbolic dynamics is used to abstract the information contained in raw data. To accommodate a layered framework for risk factors, symbolic dynamics operators were discussed. To analyze the relationships between risk factors in a layered structure, quantitative correlation values were obtained using the Kullback-Leibler distance and Kolmogorov-Sinai entropy in the symbol distribution analysis. A method for merging risk factors using the symbolic dynamics operators that enables the management of risks with multiple granularities was discussed. Finally, the method was demonstrated using an example from the Sanya distribution network.

## Supporting Information

S1 Raw Data (XLS)

S2 Raw Data (XLS)
